# The Relationship Between Vasoproliferative Tumor and Uveitis in a Multiple Sclerosis Patient: A Case Report and Review of the Literature

**DOI:** 10.4274/tjo.galenos.2019.50460

**Published:** 2019-12-31

**Authors:** Onur Özalp, Eray Atalay, Mustafa Değer Bilgeç, Nazmiye Erol, Nilgün Yıldırım

**Affiliations:** 1 Eskişehir Osmangazi University Faculty of Medicine, Department of Ophthalmology, Eskişehir, Turkey

**Keywords:** Vasoproliferative tumor, cryotherapy, uveitis, multiple sclerosis

## Abstract

Vasoproliferative retinal tumor (VPRT) is a rare, benign lesion with a variable clinical course depending on the individual. Favorable outcomes are obtained with early diagnosis and treatment of patients with VPRT. In this case report, we present a case of concomitant VPRT and multiple sclerosis along with our management of uveitis and secondary glaucoma that presumably developed following cryotherapy for VPRT.

## Introduction

Vasoproliferative retinal tumors (VPRTs) are rare, benign tumoral lesions whose pathogenesis has not been fully explained.^1^ These lesions, which appear as elevated, yellowish-pink, vascularized masses on fundus examination, are frequently located in the pre-equatorial or equatorial region of the inferior retina, especially in the 5-7 o’clock segment.^2^

VPRTs can occur as primary (74%) or secondary (26%) tumors, whit primary tumors usually being solitary while secondary tumors may be multiple.^1^ Secondary tumors may occur in ocular pathologies such as intermediate uveitis, retinitis pigmentosa, Coats disease, neurofibromatosis, retinopathy of prematurity, and familial exudative vitreoretinopathy.^1^^,3^

VPRTs can cause intraretinal or subretinal hemorrhage, vitreous hemorrhage, intraretinal or subretinal exudation, and hyperpigmentation of the retinal pigment epithelium.^2^ These sight-limiting conditions can be prevented with early diagnosis of VPRT and appropriate treatment.^2^

The purpose of this article is to present a case report of concomitant VPRT and multiple sclerosis along with our management of uveitis and secondary glaucoma that presumably developed following cryotherapy for VPRT.

## Case Report

**Figure 1:**
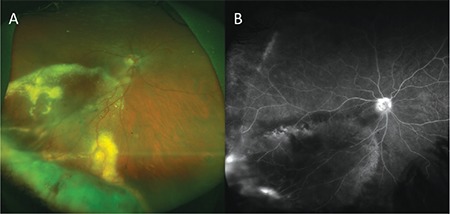
From the patient’s initial presentation: A) color fundus photograph shows vasoproliferative retinal tumor and exudation; B) fundus fluorescein angiography reveals a hyperfluorescent area in the region corresponding to the raised lesion field in the inferotemporal region, leakage from peripheral vessels, and hyperfluorescence at the optic discs

**Figure 2:**
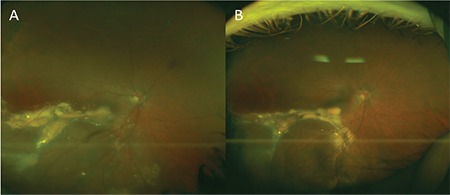
Appearance of vasoproliferative retinal tumor in color fundus photograph taken 3 months after the first cryotherapy; B) color fundus photograph at 3 months after the second cryotherapy shows the vasoproliferative retinal tumor is reduced in size

A 33-year-old man who presented with low vision in his right eye lasting 6 days was referred to our clinic from another center with the suspicion of serous retinal detachment. His history revealed that he had been diagnosed with MS 7 years earlier and had been treated with intramuscular interferon beta 1a therapy [Avonex®, 30 µg (6 million IU) once a week] for 2 years at another center. After developing side effects to the drug, he was followed without medical treatment. His best corrected visual acuity (BCVA) was measured as 0.1 in the right eye and 1.0 in the left eye with Snellen chart. The anterior segment was normal in both eyes on slit-lamp examination. Intraocular pressure (IOP) was measured as 14 mmHg bilaterally using a pneumotonometer. Fundus examination revealed a raised pink mass and severe exudation in the inferior retina of the right eye and sporadic atrophic chorioretinal areas in the left eye (Figure 1A). Optical coherence tomography (OCT) revealed serous detachment, brush border pattern, and an epiretinal membrane over the fovea. Fundus fluorescein angiography showed a hyperfluorescent area in the inferotemporal periphery consistent with the raised lesion base, ponding associated with exudative detachment extending to the macula, leakage of the peripheral vessels, and hyperfluorescence of the optic disc in the right eye, while the left eye appeared normal (Figure 1B). The patient was diagnosed with VPRT and underwent cryotherapy using the double freeze technique. His BCVA 3 months after cryotherapy was counting fingers (CF) from 3 meters. Fundus examination revealed hard exudates and a mass in the inferior temporal region (Figure 2A) and OCT revealed subretinal fluid. As additional treatment, he underwent triple-freeze cryotherapy with simultaneous intravitreal anti-VEGF injection. At follow-up 3 months after treatment, his BCVA had improved to 0.1, fundus examination revealed that the mass had shrunk (Figure 2B), and OCT showed that the epiretinal membrane persisted but the subretinal fluid had resolved.

**Figure 3:**
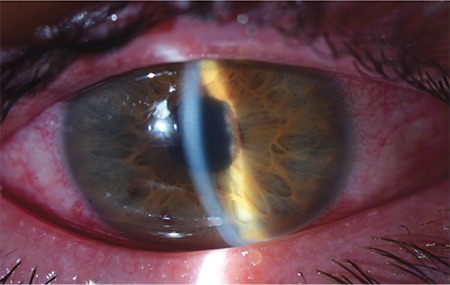
Anterior segment photograph of the patient at presentation for uveitic glaucoma shows ciliary injection, iris bombe, seclusio pupillae, and corneal edema

At 6 months after the last cryotherapy, the patient presented to the emergency department with pain in his right eye. His BCVA was again CF at 3 m in the right eye and 1.0 in the left eye. Slit-lamp examination revealed ciliary injection, cells in the anterior chamber (+++), iris bombe seclusio pupillae, and corneal edema in the right eye (Figure 3). The anterior segment of the left eye was normal. IOP measured by applanation tonometry was 35 mmHg in the right eye and 15 mmHg in the left eye. The patient was hospitalized with the suspicion of secondary angle-closure glaucoma. Two peripheral laser iridotomies were performed at the 1 and 11 o’clock positions. Topical mydriatic, antiglaucomatous, corticosteroid drops along with oral acetazolamide were initiated. Due to persistently elevated IOP during follow-up, trabeculectomy with 5-fluorouracil was performed. After 1 week, his IOP was 30 mmHg, so argon laser suturolysis was performed. The next day, his IOP was decreased to 20 mmHg. In his follow-up visit 2 months after trabeculectomy, visual acuity in the right eye was CF at 3 m, IOP was 20 mmHg, and slit-lamp examination revealed a functioning bleb, clear cornea, and deepened anterior chamber.

## Discussion

Although it has no pathognomonic finding, suspicion of VPRT should arouse when a solitary mass with yellowish-pink appearance and vascularization is observed on fundus examination.^2^ It is commonly observed between the ages of 40 and 60 years, with no significant difference in prevalence between men and women.^1^ In a retrospective study, it was observed that VPRTs were most commonly located in the inferotemporal (42%) segment, followed by inferior (21%) and temporal (15%).^1^

It has been reported that primary VPRTs emerge at a later age, are often solitary and unilateral, and cause fewer symptoms.^4^ In a retrospective study by Shields et al.^1^, secondary VPRTs were most commonly associated with intermediate uveitis (28%), retinitis pigmentosa (21%), toxoplasma retinitis (7%), toxocariasis (7%), and traumatic chorioretinopathy (7%). When pediatric patients were evaluated, secondary VPRTs were again most often associated with intermediate uveitis.^5^

Intermediate uveitis is an ocular inflammatory syndrome characterized by minimal anterior segment reaction and inflammatory cells and debris in the vitreous, and is often observed in children and young adults.^6^ In a study by Ness et al.^7^ including 159 cases of intermediate uveitis, most cases were idiopathic (58.5%), while MS was the leading known cause (19.5%). Other studies have also examined systemic diseases in patients with intermediate uveitis, with the prevalence of MS reported as 7% by Boskovich et al.^8^ and 11% by Raja et al.^9^ The relationship between MS and uveitis remains unclear; in one study evaluating MS patients, the prevalence of uveitis was found to be 0.52%, with intermediate and panuveitis being common forms.^10^ In the same study, evaluation of complications in patients with intermediate uveitis showed that cataracts were most common, and glaucoma and retinal neovascularization were also observed.^10^ In another study of patients with intermediate uveitis, the most common complications were cystoid macular edema, cataracts, and posterior synechia, and glaucoma was also noted.^11^

The pathogenesis of secondary VPRT associated with intermediate uveitis is believed to involve a reactive process against factors released into the environment with the emergence of uncontrolled proliferation of fibrous tissue and angiogenesis in the retina secondary to disruption of the blood-retina barrier.^2^ On the other hand, it has also been suggested that the presence of intraocular inflammation and uveitis may be due to a reactive, or “spillover” phenomenon in which inflammatory cells leak from tumoral lesion vessels into the vitreous.^2^

If VPRTs are asymptomatic, they can be monitored without treatment. In symptomatic cases, treatment may involve photocoagulation, cryotherapy, laser brachytherapy, radiotherapy, photodynamic therapy, or anti-VEGF therapy. Vitreoretinal surgery can be performed in patients with vitreous hemorrhage.^1^^,3,12,13,14,15^

Of 160 patients with retinal detachment who underwent simultaneous cryotherapy and detachment surgery, 19 (12%) developed postoperative pigment shedding, while 7 (12%) of the 60 patients who did not undergo subretinal fluid drainage developed postoperative uveitis.^16^ The same study reported that post-cryotherapy uveitis occurred in an average of 5-8 days.^16^ Although our patient presented with uveitis 6 months after the last cryotherapy, we do not rule out the possibility that uveitis may have developed as a complication of cryotherapy.

In conclusion, although VPRTs can occur secondary to uveitis, they may also be a cause of uveitis. However, it should be kept in mind that cryotherapy for VPRT may also be associated with uveitis and the complications of uveitis. Optic neuritis and retinal periphlebitis, which are among the most important signs of MS uveitis, are sometimes the findings that lead to a diagnosis.^17^^,18^ The leakage from the peripheral retinal vessels and optic disc staining initially observed in our patient may have been associated with his existing MS. However, the subsequent, more pronounced presentation of uveitis is believed to have been associated with cryotherapy. MS should be kept in mind with young patients for whom other causes of secondary VPRT cannot be established.
